# Understanding Uptake and Experience of Interpreting Services in Primary Care in a South Asian Population in the UK

**DOI:** 10.1001/jamanetworkopen.2022.44092

**Published:** 2022-11-29

**Authors:** Katriina L. Whitaker, Demi Krystallidou, Emily D. Williams, Georgia Black, Cecilia Vindrola-Padros, Paramjit Gill, Sabine Braun

**Affiliations:** 1 School of Health Sciences, University of Surrey, Guildford, United Kingdom; 2 Centre for Translation Studies, University of Surrey, Guildford, United Kingdom; 3 Department of Applied Health Research, University College London, London, United Kingdom; 4 Wolfson Institute of Population Health, Queen Mary University of London, London, United Kingdom; 5 Department of Targeted Intervention, University College London, London, United Kingdom; 6 Division of Health Sciences, Warwick Medical School, University of Warwick, Coventry, United Kingdom

## Abstract

This cross-sectional study addresses the evidence gap in uptake of interpretation services and patient experiences among a South Asian population in the UK without English language proficiency.

## Introduction

Addressing language barriers in accessing health care may improve equitable access in line with current United Nations Sustainable Development Goals.^1^ English proficiency is associated with socioeconomic position, social segregation, and employment,^2^ and the intersectionality of ethnicity, immigration status, and lack of language proficiency results in cumulative disadvantage.^3^ Guidance for commissioners in the UK states that language and communication requirements should not prevent patients from receiving equitable care.^4^ Limited evidence is available on interpreting service uptake and patient experience that is crucial to ensure services reduce ethnic and socioeconomic health inequalities.^5^ We aimed to address this evidence gap.

## Methods

This national, cross-sectional community-based pilot survey conducted from December 1, 2020, to January 5, 2021, adhered to the STROBE reporting guideline. Ethical approval was obtained from the University of Surrey. Survey interviews were conducted by telephone by multilingual researchers, and participants provided verbal informed consent. Eligibility criteria included self-reported limited or no English language proficiency, age older than 18 years, and self-reported Pakistani, Indian, or Bangladeshi ethnicity. Convenience and snowball sampling were undertaken to identify eligible participants across the UK, including London, Birmingham, Leicester, Manchester/Oldham, and Bradford. Measures included type(s) of interpreting service used and perceived barriers to their uptake. We evaluated differences between people who had and had not used interpreting services with χ^2^ and Fisher exact tests. Two-sided *P* < .05 indicated statistical significance. Analyses were performed using SPSS, version 28.0.1.0 (IBM Corporation).

## Results

Of 105 people in the sample, 35 (33.3%) each reported Indian, Bangladeshi, or Pakistani ethnicity, with ages ranging from 18 to 79 years. Fifty-four participants (51.4%) were women and 51 (48.6%) were men; 83 (79.0%) were married or cohabiting; and 17 (16.2%) had no formal education. Sixty-three participants (60.0%) reported using at least 1 type of formal interpreting service, including face-to-face (57 [54.3%]), telephone (18 [17.1%]), and video-mediated (5 [4.8%]). Forty-seven participants (44.8%) reported family or friends interpreting for them during consultations; of these, only 18 (38.3%) reported formal interpreting service uptake. Thirty-four participants (32.4%) reported having a physician or nurse who speaks their language; of these, 11 (32.4%) used a formal interpreting service. Thirty-seven participants (35.2%) reported being offered a choice of language support by primary care clinicians. Compared with participants who had never used formal interpreting services, those who had were more likely to have no formal education (16 of 63 [25.4%] vs 1 of 42 [2.4%]), report lower confidence in managing conditions (24 of 63 [38.1%] vs 7 of 42 [16.7%]), perceive a need for language support (51 of 63 [81.0%] vs 16 of 42 [38.1%]), and have been told about language support by primary care clinicians (35 of 63 [55.6%] vs 12 of 42 [28.6%]) (Table). The Figure summarizes interpreting service barriers.

**Table.** Use of Formal Interpreting Services by Sociodemographic Characteristics

Characteristic	Participants^a^			*P* value
	Total (N = 105)	Have used formal interpreting services (n = 63)	Have never used formal interpreting services (n = 42)	
Sex				
Women	54 (51.4)	33 (52.4)	21 (50.0)	.84
Men	51 (48.6)	30 (47.6)	21 (50.0)	
Age, y^b^				
18-24	6 (5.7)	3 (4.8)	3 (7.1)	.07
25-34	16 (15.2)	9 (14.3)	7 (16.7)	
35-44	43 (41.0)	23 (36.5)	20 (47.6)	
45-54	21 (20.0)	13 (20.6)	8 (19.0)	
55-64	13 (12.4)	10 (15.9)	3 (7.1)	
65-74	4 (3.8)	3 (4.8)	1 (2.4)	
75-79	2 (1.9)	2 (3.2)	0	
Ethnicity				
Bangladeshi	35 (33.3)	23 (36.5)	12 (28.6)	.62
Indian	35 (33.3)	19 (30.2)	16 (38.1)	
Pakistani	35 (33.3)	21 (33.3)	14 (33.3)	
Educational level^c^				
No formal education	17 (16.2)	16 (25.4)	1 (2.4)	.002
Primary school	31 (29.5)	18 (28.6)	13 (31.0)	
Secondary school	33 (31.4)	19 (30.2)	14 (33.3)	
College or sixth form	13 (12.4)	5 (7.9)	8 (19.0)	
University level	3 (2.9)	2 (3.2)	1 (2.4)	
Prefer not to say	8 (7.6)	3 (4.8)	5 (11.9)	
Living arrangements^d^				
Own home outright	8 (7.6)	6 (9.5)	2 (4.8)	.83
Own home with mortgage	25 (23.8)	13 (20.6)	12 (28.6)	
Rent from local authority	28 (26.7)	17 (27.0)	11 (26.2)	
Rent privately	29 (27.6)	17 (27.0)	12 (28.6)	
Other (eg, live with family)	12 (11.4)	8 (12.7)	4 (9.5)	
Prefer not to say	3 (2.9)	2 (3.2)	1 (2.4)	
Relationship status^e^				
Married or cohabiting	83 (79.0)	50 (79.4)	33 (78.6)	.80
Single or never married	12 (11.4)	7 (11.1)	5 (11.9)	
Widowed	6 (5.7)	5 (7.9)	1 (2.4)	
Divorced or separated	2 (1.9)	1 (1.6)	1 (2.4)	
Prefer not to say	2 (1.9)	0	2 (4.8)	
Spoken language				
Bengali	35 (33.3)	23 (36.5)	12 (28.6)	.33
Gujrati	3 (2.9)	3 (4.8)	0	
Hindi	7 (6.7)	5 (7.9)	2 (4.8)	
Punjabi	42 (40.0)	21 (33.3)	21 (50.0)	
Urdu	18 (17.1)	11 (17.5)	7 (16.7)	
Religion				
Hindu	12 (11.4)	9 (14.3)	3 (7.1)	.25
Muslim	71 (67.6)	44 (69.8)	27 (64.3)	
Sikh	21 (20.0)	10 (15.9)	11 (26.2)	
Prefer not to say	1 (1.0)	0	1 (2.4)	
Country of birth				
Outside of UK	103 (98.1)	61 (96.8)	42 (100.0)	.24
UK	2 (1.9)	2 (3.2)	0	
Close family nearby				
Yes	77 (73.3)	48 (76.2)	29 (69.0)	.52
No	26 (24.8)	13 (20.6)	13 (31.0)	
Prefer not to say	2 (1.9)	2 (3.2)	0	
English language proficiency				
Do not speak English well	94 (89.5)	54 (85.7)	40 (95.2)	.19
Do not speak English at all	11 (10.5)	9 (14.3)	2 (4.8)	
Self-rated health^f^				
Poor	15 (14.3)	11 (17.5)	4 (9.5)	>.99
Fair	44 (41.9)	24 (38.1)	20 (47.6)	
Good	37 (35.2)	24 (38.1)	13 (31.0)	
Very good	9 (8.6)	4 (6.3)	5 (11.9)	
No. of primary care visits in past 12 mo^g^				
0	8 (7.6)	3 (4.8)	5 (11.9)	.26
1	34 (32.4)	21 (33.3)	13 (31.0)	
2	37 (35.2)	25 (39.7)	12 (28.6)	
≥3	26 (24.8)	14 (22.2)	12 (28.6)	
Comorbidities^h^				
Circulation problems	8 (7.6)	5 (7.9)	3 (7.1)	.79
Breathing problems	9 (8.6)	4 (6.3)	5 (11.9)	
Arthritis	11 (10.5)	6 (9.5)	5 (11.9)	
Depression or anxiety	8 (7.6)	4 (6.3)	4 (9.5)	
Diabetes	27 (25.7)	17 (27.0)	10 (23.8)	
Heart problems	3 (2.9)	3 (4.8)	0	
High blood pressure	20 (19.0)	14 (22.2)	6 (14.3)	
High cholesterol level	27 (25.7)	17 (27.0)	10 (23.8)	
Kidney problems	1 (1.0)	1 (1.6)	0	
Stroke	1 (1.0)	0	1 (2.4)	
Other	33 (31.4)	20 (31.7)	13 (31.0)	
Prefer not to say	20 (19.0)	12 (19.0)	8 (19.0)	
Confidence in managing conditions				
Not confident (not at all/not very)	31 (29.5)	24 (38.1)	7 (16.7)	.02
Confident (fairly/very)	64 (61.0)	34 (54.0)	30 (71.4)	
Do not know	6 (5.7)	3 (4.8)	3 (7.1)	
NA	4 (3.8)	2 (3.2)	2 (4.8)	
Disability				
No	101 (96.2)	60 (95.2)	41 (97.6)	.69
Yes	3 (2.9)	2 (3.2)	1 (2.4)	
Do not know	1 (1.0)	1 (1.6)	0	
Perceived need for language support				
No	4 (3.8)	1 (1.6)	3 (7.1)	<.001
No, my physician or nurse speaks my native language	34 (32.4)	11 (17.5)	23 (54.8)	
Yes	67 (63.8)	51 (81.0)	16 (38.1)	
Told about language support				
No	31 (29.5)	12 (19.0)	19 (45.2)	.007
I am not sure	27 (25.7)	16 (25.4)	11 (26.2)	
Yes	47 (44.8)	35 (55.6)	12 (28.6)	

Abbreviation: NA, not applicable.

^a^ Data are presented as No. (%) of participants. Percentages have been rounded and may not total 100.

^b^ Collapsed for comparison of those 55 years and older vs those younger than 55 years.

^c^ Collapsed for comparison of those with any level of education vs those with no formal education.

^d^ Collapsed for comparison of those owning their own home outright or with a mortgage vs those not owning their own home.

^e^ Collapsed for comparison of those married or cohabitating vs other relationship status.

^f^ Collapsed for comparison of those with poor or fair health vs those with good or very good health.

^g^ Collapsed for comparison of those with at least 1 visit vs those with no visits.

^h^ Participants may report more than 1 comorbidity. *P* value calculated as 0 vs at least 1.

**Figure.**
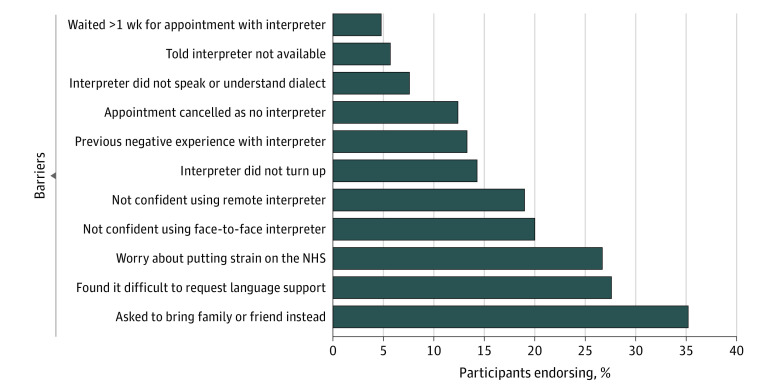
Potential Barriers to Using Formal Interpreting Services NHS indicates National Health Service.

## Discussion

This cross-sectional survey study found that most respondents reported using at least 1 type of formal interpreting service, with face-to-face interpreting being most common, followed by telephone interpreting. Video-mediated interpreting use was rare. However, nearly half of the respondents relied on family or friends. Raising awareness of professional interpreting services, patient education, and addressing perceived barriers to accessing formal language support services have the potential to improve access among groups who lack English proficiency.

Our study has some limitations. Data were collected during the COVID-19 pandemic, which may have affected responses, although we did not restrict responses to this timescale, and some likely related to prepandemic experiences. Although we found important indications about the likely influences on interpreting service uptake, larger-scale studies are required to account for the selection bias associated with snowball sampling.^6^

Use of formal interpreters is known to close gaps in quality of clinical care for patients with limited English proficiency. Our survey, which was developed to understand why uptake and experiences may vary, can be used at scale to obtain this vital information to improve equitable health service access.
